# Customized Frozen Embryo Transfer after Identification of the Receptivity Window with a Transcriptomic Approach Improves the Implantation and Live Birth Rates in Patients with Repeated Implantation Failure

**DOI:** 10.1007/s43032-020-00252-0

**Published:** 2020-07-28

**Authors:** Delphine Haouzi, Frida Entezami, Antoine Torre, Charlène Innocenti, Yannick Antoine, Charlotte Mauries, Claire Vincens, Sophie Bringer-Deutsch, Anna Gala, Alice Ferrieres-HOA, Jeanine Ohl, Beatriz Gonzalez Marti, Sophie Brouillet, Samir Hamamah

**Affiliations:** 1 Univ Montpellier, INSERM U1203, EmbryoPluripotency, Montpellier, France; 2 IRMB, Univ Montpellier, INSERM, Montpellier, France; 3 CHU Montpellier, ART/PGD Department, Arnaud de Villeneuve Hospital, Montpellier, France; 4 ART Department, American Hospital of Paris, Neuilly-Sur-Seine, France; 5 Laboratoire Eylau-UNILABS-La Muette, Clinique de La Muette-Ramsay-Générale de Santé, Paris, France; 6 Division of Child Health, Obstetrics & Gynaecology Department, University of Nottingham, Nottingham, UK; 7 Department of reproductive medicine, CMCO, Schiltigheim, France; 8 Univ Grenoble-Alpes, INSERM U1036, Commissariat à l’énergie atomique et aux énergies alternatives (CEA), Institut de Biosciences et Biotechnologies de Grenoble (BIG), Laboratoire Biologie du Cancer et de l’Infection (BCI), 38000 Grenoble, France

**Keywords:** Transcriptome, Win-Test, Implantation window, Receptivity window

## Abstract

The aim of this prospective study was to evaluate outcome benefits expected in repeated implantation failure (RIF) patients (*n* = 217) after customized embryo transfer based upon identification of the receptivity window by transcriptomic approach using the Win-Test. In this test, the expression of 11 endometrial genes known to be predictive of endometrial receptivity is assessed by RT-PCR in biopsies collected during the implantation window (6–9 days after the spontaneous luteinizing hormone surge during natural cycles, 5–9 days after progesterone administration during hormone replacement therapy cycles). Then, patients underwent either customized embryo transfer (cET, *n* = 157 patients) according to the Win-Test results or embryo transfer according to the classical procedure (control group, *n* = 60). Pregnancy and live birth rates were compared in the two groups. The Win-Test showed that in 78.5% of women, the receptivity window lasted less than 48 h, although it could be shorter (< 24 h, 9.5%) or longer (> 48 h, 12%). This highlighted that only in 20% of patients with RIF the endometrium would have been receptive if the classical embryo transfer protocol was followed. In the other 80% of patients, the receptivity window was delayed by 1–3 days relative to the classical timing. This suggests that implantation failure could be linked to inadequate timing of embryo transfer. In agreement, both implantation (22.7% vs. 7.2%) and live birth rates per patient (31.8% vs. 8.3%) were significantly higher in the cET group than in the control group. cET on the basis of the Win-Test results could be proposed to improve pregnancy and live birth rates.

ClinicalTrials.gov ID: NCT04192396; December 5, 2019, retrospectively registered.

**Electronic supplementary material:**

The online version of this article (10.1007/s43032-020-00252-0) contains supplementary material, which is available to authorized users.

## Introduction

Infertility is a major public health problem that affects more than one in six couples of childbearing age. According to a recent French national perinatal survey and the French epidemiological observatory for fertility, almost 40% of couples do not conceive after 1 year of regular sexual intercourses without contraception [1, 2]. Moreover, the average live birth rate after assisted reproductive technology (ART), irrespective of age and indication, is low (< 20%) [3]. Despite the many ART advances, embryo implantation rate remains problematically low. Implantation failure is mainly correlated with embryo competency and lack of uterine receptivity leading to altered embryo–endometrium synchronization. Several non-invasive parameters have been proposed to assess endometrial receptivity and the “implantation window” timing, such as endometrium thickness, endometrial morphology by ultrasound assessment, and endometrial and sub-endometrial blood flow measure by Doppler ultrasound [4, 5]. However, these approaches have given controversial results and their positive predictive value for endometrial receptivity evaluation is low [5, 6]. Yet, it is crucial to exactly determine the endometrial receptivity occurrence and duration. Transcriptomic and proteomic approaches also have been used to define molecular signatures and identify specific biomarkers of human endometrial receptivity (comprehensive review in [7]). Yet, very few molecular diagnostic tools are available to characterize the implantation window [8, 9]. Using our transcriptomic data, we previously identified a set of genes (*BCL2L10*, *CD68*, *TRPC4*, *SORCS1*, *FST*, *KRT18*, *LAMB3*, *MFAP5*, *ANGPTL1*, *PROK1*, and *C2CD4B*) that are overexpressed in the endometrium during the implantation window and that seem to be relevant candidate biomarkers of human endometrial receptivity [7, 10–14]. After validation of these transcriptomic results by reverse transcription-quantitative PCR (RT-qPCR), we tested these candidate endometrial receptivity biomarkers in fertile patients and in an ex vivo model (i.e., stromal and epithelial endometrium cells) [10, 14, 15]. We then developed an innovative test based on the quantification by RT-PCR of 11 of these genes in endometrium biopsies that we called Win-Test for Window Implantation Test [16]. The strength and robustness of the Win-Test have been extensively reviewed in [7]. However, the Win-Test clinical benefit in terms of pregnancy and live birth rates has never been established. Therefore, the aim of this prospective interventional multicenter study was to determine the ART outcomes (pregnancy and live birth rates) after frozen embryo transfer according to the Win-Test results compared with the usual procedure.

## Materials and Methods

### Study Design and Sample Collection

Between January 2015 and June 2018, 217 women (mean ± SD, age 37.04 ± 4.4 years) were recruited after signature of the written informed consent and approval by the IRB of the Montpellier University Hospital. All recruited women had a history of repeated implantation failure (RIF), according to the definition by Polanski et al. [17] (mean ± SD, number of previous failed attempts 4.58 ± 2.15), after fresh and/or frozen-thawed embryo transfer. This sample included also patients who benefited from oocyte/embryo donation (*n* = 41). Patients were referred for female (44.9%), male (23.4%), idiopathic (21%), and mixed (10.7%) infertility. All underwent a classical infertility evaluation that included transvaginal sonography and uterine cavity assessment by hysteroscopy. In addition, the following data were recorded after each implantation failure (thrombophilia, coagulation factors, immunologic response, and thyroid function). All patients were scheduled for cryopreserved embryo transfer during a hormone replacement therapy (HRT) cycle with (*n* = 44 patients) or without (*n* = 111) GnRH analogue (GnRHa), or during a natural cycle (*n* = 62).

All patients underwent endometrial receptivity estimation with the Win-Test during the theoretical implantation window between days 5 and 9 after progesterone treatment start (Pg + 5 to Pg + 9) or between day 6 and day 9 after luteinizing hormone surge (LH + 6 to LH + 9) [10, 15]. Biopsy was rinsed in PBS, then placed in a cryotube containing lysis buffer (RLT; Qiagen), and quickly frozen at minimum − 80 °C (dry ice or liquid nitrogen) until shipment in dry ice to our Montpellier ART center.

**Fig. 1:**
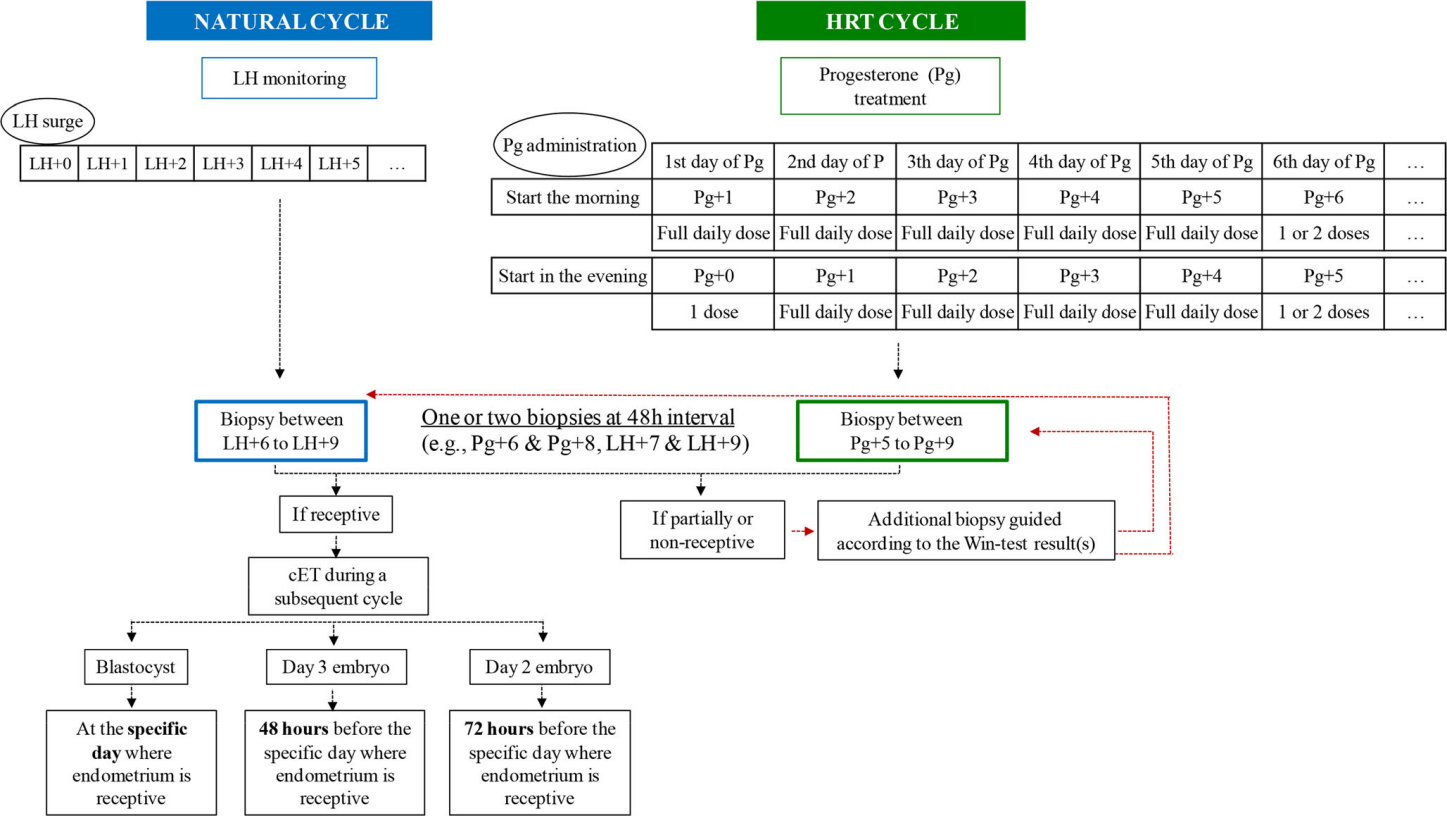
Outline of the Win-Test procedure. HRT, hormone replacement therapy; LH, luteinizing hormone; Pg, progesterone; cET, customized embryo transfer

According to each center protocol, the HRT regimen involved estradiol administration (fixed dose of 4 to 6 mg/day of estrogen; or a progressively increasing dose from 2 mg to a maximum of 6 mg daily, changing dose every 4 days) through the oral route and/or transdermal patch starting from day 1 or 2 of the menstrual cycle until progesterone administration. The final endometrial maturation was obtained with 600 to 800 mg progesterone per day. Some patients received a subcutaneous/intramuscular injection of triptorelin (3 mg) at day 21 of the previous menstrual cycle. The endometrium ultrasound pattern and thickness were assessed between days 12 and 14 after menses and progesterone was administered when endometrium showed a trilaminar pattern and a thickness > 7 mm. The number of progesterone treatment days was calculated according to the daily progesterone dose (1 day of complete dose corresponded to Pg + 1) (Fig. 1).

For patients on natural cycle, estradiol, progesterone, and LH were quantified in serum to determine the LH surge. Specifically, the exact time of spontaneous ovulation was determined retrospectively on the basis of (1) baseline serum LH and progesterone concentration at days 1–3 of the cycle; (2) serum estradiol, progesterone (< 1.5 ng/ml), and LH concentration from day 10 of the cycle to the LH surge (up to daily quantification, if required; serum LH level increase by more than threefold compared with the baseline value); and (3) serum progesterone increase to more than 1.5 ng/ml after the LH surge. The day of LH surge was considered as day LH + 0 (Fig. 1).

### The Win-Test

The 11 genes involved in human endometrial receptivity included in the Win-Test were selected by comparing the gene expression profiles of receptive (LH + 7, *n* = 31) and pre-receptive (LH + 2, *n* = 31) endometrium obtained in our previous transcriptomic analysis [10]. Specifically, the Significant Analysis of Microarrays (SAM; Stanford University, USA [18]) and *t* test were used to select genes with an absolute fold-change >2 and a false discovery rate < 0.05. Concomitantly, class prediction applied to microarray experiments was used to identify biomarkers that are putatively involved in endometrial receptivity [10, 19]. The 11 most upregulated genes identified by both approaches were included in the Win-Test [7, 10–13]. Each test includes a receptive endometrium (LH + 7) and a pre-receptive endometrium (LH + 2) sample as positive and negative control, respectively. Each gene of the Win-Test must be overexpressed compared with a pre-receptive endometrium to define the endometrium as “receptive.” As the microarray data clearly showed that the expression of the 11 genes might vary among patients, threshold values of their mean of expression were defined to take into account this inter-patient variability. Accordingly, an endometrium is defined as “receptive” when the mean expression of the 11 genes is ≥ 70%, “partially receptive” when between 50 and 70%, and “non-receptive” when < 50% of the expression level of the positive control which is meant to be 100% and the negative control < 15%. The Win-Test results are provided within 5 days post-reception of biopsies.

The Win-Test performance to predict pregnancy outcome using receiver operating characteristics analysis was evaluated in the first RIF patients who underwent customized embryo transfer (cET) as described in [20] (Supplementary Fig. S1).

### Customized Frozen Embryo Replacement According to the Win-Test Results

Our strategy consisted in performing cET of blastocysts based on the endometrium receptivity day identified using the Win-Test. Therefore, frozen day 2 or day 3 embryos were transferred 72 or 48 h before this day, respectively. When the endometrial sample was defined as non-receptive or partially receptive by the Win-Test, a second evaluation was performed subsequently according to the first Win-Test result (Fig. 1).

Positive pregnancy test was defined as a positive β-hCG serum concentration followed by at least two increasing β-hCG values at 48-h interval, to exclude early biochemical pregnancies. Clinical pregnancy was defined by the ultrasound visualization of a gestational sac with embryo heartbeat. The implantation rate (i.e., the number of observed gestational sacs relative to the total number of transferred embryos) and live birth rate (i.e., the birth of at least one live baby after more than 24 weeks of amenorrhea) were also recorded after cET according to the Win-Test results. In the control group, embryo transfer was performed as usually routine protocol (i.e., at Pg + 5/Pg + 6 for HRT cycles and at LH + 6/LH + 7 for natural cycles).

### Quantitative RT-PCR Analyses

For the Win-Test, 0.5 μg RNA from each endometrial sample was used for RT-qPCR analysis according to the manufacturer’s recommendations (Applied Biosystems, Villebonsur Yvette, France), as previously described [15, 16]. For qPCR, 2 μl of first strand DNA (1:5 dilution) was added to a 10-μl reaction mixture containing 0.25 μM of each primer and 5 μl of 2× LightCycler 480 SYBR Green I Master mix (Roche, Mannheim, Germany). DNA was amplified for 45 cycles with an annealing temperature of 63 °C using the Light Cycler 480 detection system (Roche). Gene expression values were normalized to the expression of three housekeeping genes, hypoxanthine phosphoribosyltransferase 1 (*HPRT1*), glyceraldehyde-3-phosphate dehydrogenase (*GAPDH*), and phosphoglycerate kinase 1 (*PGK1*) using the following formula: *E*_tested gene_^ΔCt^/*E*_housekeeping gene_^ΔCt^ (*E* = 10^−1/slope^), ΔCt = Ct control − Ct unknown, where *E* corresponds to the PCR reaction efficiency. The *E* value was obtained using a standard curve that varies in function of the used primers. One receptive endometrium sample from a patient in natural cycle (LH + 7) was used as positive control and a non-receptive sample (LH + 2) as negative control. Each sample was analyzed in duplicate and multiple water blanks were included.

### Statistical Analyses

Statistical analyses were performed with the GraphPad Prism 8 software. Data are expressed as the mean ± SD and differences between groups were considered significant when the Student’s *t* test gave a *P* value < 0.05. ROC curve analysis was performed with the MedCalc software, according to the methodology described by DeLong et al. [21].

## Results

### The Receptivity Window Duration Is Patient Dependent

**Fig. 2:**
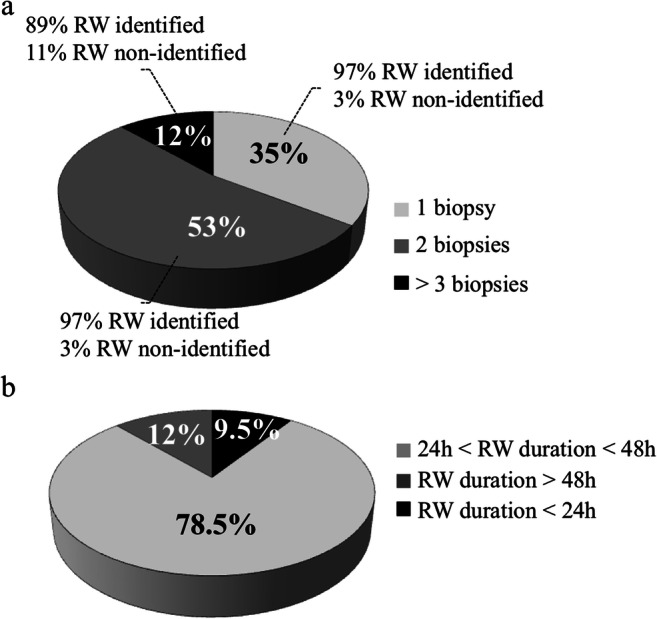
**a** Percentage of patients with RIF in whom the endometrial receptivity window (RW) was or was not identified in function of the number of endometrial biopsy performed. **b** RW duration in patients with RIF who had at least two endometrial biopsies

In total, 419 endometrial biopsies from 217 patients with RIF were analyzed: 51.1% of patients (*n* = 111) were in HRT cycles, 20.3% (*n* = 44) in HRT with GnRHa, and 28.6% in natural cycles (*n* = 62). Most patients underwent one (35%) or two consecutive endometrial biopsies at 48-h interval (53%). Only 12% of patients needed three or more biopsies to identify the receptivity window (Fig. 2a). No more than two endometrial biopsies were performed during a single cycle, at Pg + 6 and Pg + 8, or LH + 7 and LH + 9 under HRT or natural cycles, respectively. For patients requiring more than two endometrial biopsies, at least one was performed during a subsequent cycle with the same treatment and biopsy timing was guided according to the first Win-Test result (e.g., if partially receptive at LH + 7 and non-receptive at LH + 9 the additional biopsy is recommended at LH + 8). Overall, the number of biopsies per patient was not significantly different between patients in natural and HRT cycles.

Among the patients with one endometrial biopsy (*n* = 75), the receptivity window was identified in 73 (97%). In the group of patients with two endometrial biopsies at 48-h interval (*n* = 115), the receptivity window was identified in 111 (97%) (Fig. 2a). In addition, the endometrium was considered receptive only in one of the two biopsies in 98 women (88%) and in both in 13 patients (12%). In the group of patients with three endometrial biopsies (*n* = 27), the receptivity window was identified in 24 patients (89%) (Fig. 2a).

In the group of patients who underwent two or more endometrial biopsies with successful identification of the receptivity window (*n* = 135), the receptivity window lasted 24–48 h in 106 patients (78.5%). Its duration was longer than 48 h in 16 patients (12%) and shorter than 24 h in 13 patients (9.5%) (Fig. 2b).

Whatever the receptivity window duration, the analysis of the endometrial receptivity status at different time points within the same cycle or in subsequent cycles (under the same condition/treatment) in the same patient showed that endometrial receptivity acquisition was a progressive process, as revealed by the mean expression levels of the Win-Test genes, during both natural and HRT ± GnRHa cycles with a tendency for a more progressive process under HRT. Conversely, the implantation window closure occurred rapidly, within 24 h after its detection (Supplementary Fig. S2).

### The Appearance of the Receptivity Window Is Patient Dependent

**Fig. 3:**
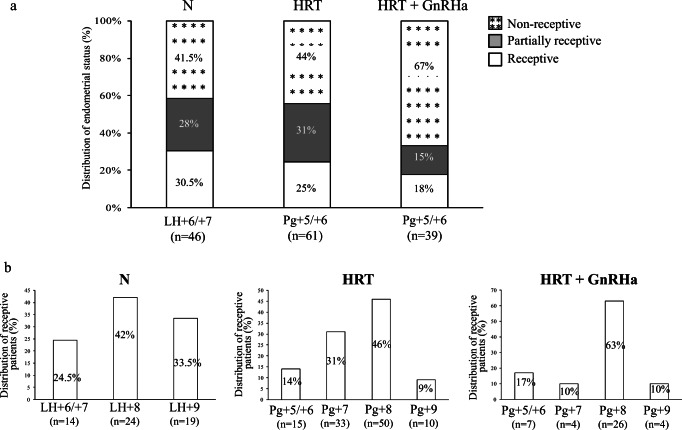
(**a**) Endometrial receptivity status according to the Win-Test performed at LH + 6/LH + 7 (natural cycles) and at Pg + 5/Pg + 6 (HRT cycles with or without GnRHa). (**b**) Percentage of patients with receptive endometrium (Win-Test) at different time points during natural and HRT cycles with or without GnRHa. GnRHa, GnRH analogue; HRT, hormone replacement therapy; LH, luteinizing hormone; N, natural cycle; Pg, progesterone

Among the 62 patients in natural cycle, the Win-Test was performed at LH + 6/LH + 7 in 46 patients. At this specific time point, only 30.5% (14/46) were receptive, while the others were either partially receptive (28%, 13/46) or non-receptive (41.5%, 19/46) (Fig. 3a). In total, the receptivity window was identified in 57/62 women (92%), and was mainly at LH + 8 (42%, 24/57), followed by LH + 6/LH + 7 (24.5%, 14/57) and LH + 9 (33.5%, 19/57) (Fig. 2b). Among the 111 patients on HRT, 61 patients were evaluated at Pg + 5/Pg + 6, and 25% were receptive (15/61), 31% partially receptive (19/61), and 44% non-receptive (27/61). The receptivity window could be identified in 108/111 patients (97%) on HRT, mainly at Pg + 7 (30.5%, 33/108) and Pg + 8 (46%, 50/108). For the remaining patients, endometrium was receptive at Pg + 5/Pg + 6 (14%, 15/108) and Pg + 9 (9%, 10/108) (Fig. 3b).

Among the 44 patients on HRT with GnRHa, 39 patients were evaluated at Pg + 5/Pg + 6. Endometrium was receptive in 26/39 (67%), partially receptive in 6/39 (15%), and non-receptive in 7/39 (18%) (Fig. 3a). In total, the receptivity window could be identified in 41/44 patients, mostly at Pg + 8 (63%; 26/41), followed by Pg + 5/Pg + 6 (17%; 7/41), Pg + 7 (4%; 4/41), and Pg + 9 (4%; 4/41) (Fig. 3b).

### Pregnancy Outcome According to the Win-Test Strategy in Patients with RIF

**Fig. 4:**
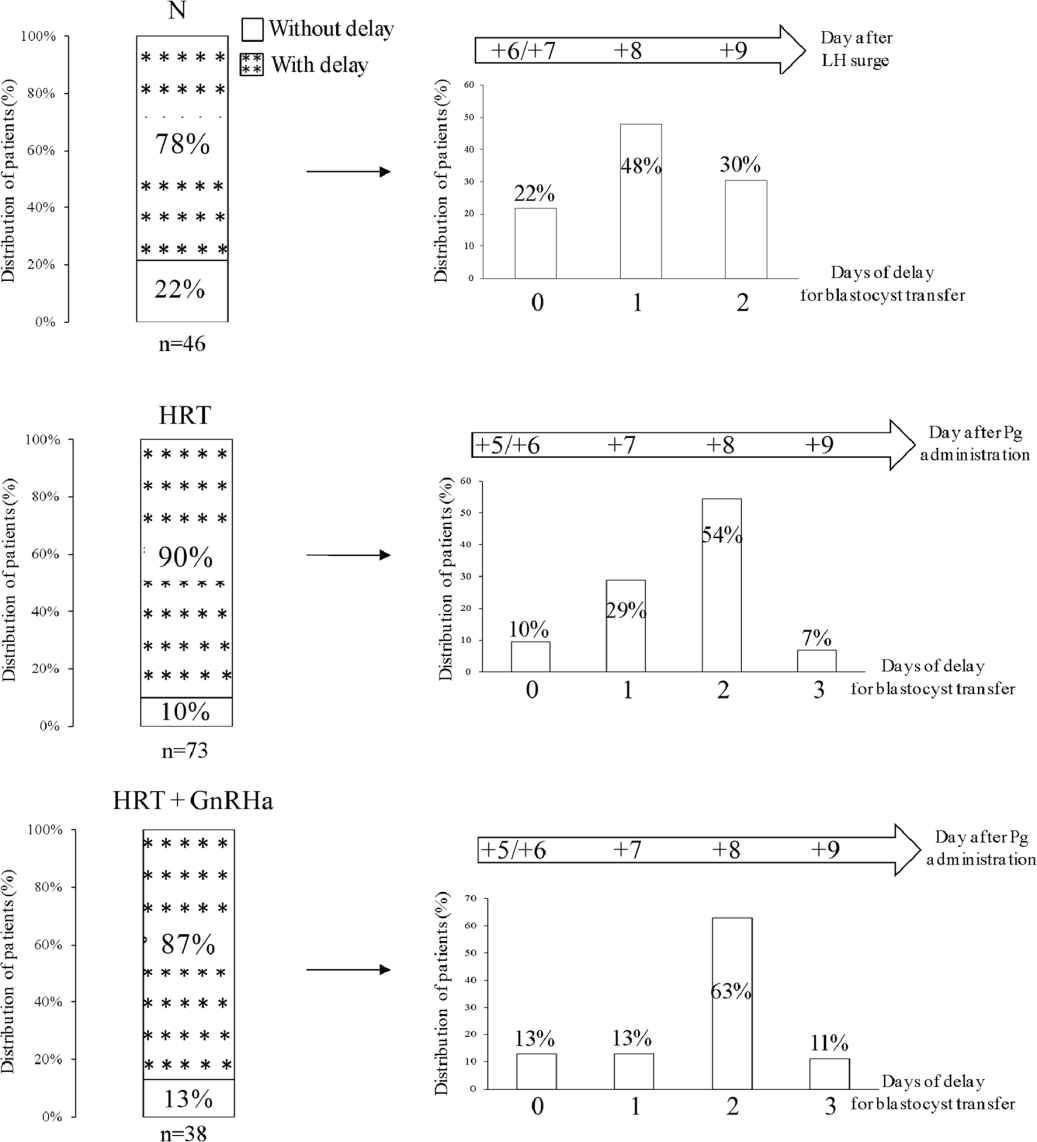
Receptivity window timing in the 157 patients with RIF (*n* = 46 in natural cycles; *n* = 73 in HRT cycles; *n* = 38 in HRT with GnRHa cycles) who underwent cET according to the Win-Test results. The receptivity window was considered delayed relative to the classical embryo transfer strategy for natural and HRT cycles. Bl, blastocyst; cET, customized embryo transfer; GnRHa, GnRH analogue; LH, luteinizing hormone; Pg, progesterone; RIF, repeated implantation failure

To evaluate the clinical suitability of the Win-Test, 157 patients underwent embryo transfer according to the Win-Test results (cET group) and 60 (control group) according to the classical protocol. The mean age (mean ± SD, 37.2 ± 4.3 vs. 36.7 ± 4.5 years, *p* = 0.47), number of previous failed attempts (mean ± SD, 4.4 ± 1.9 vs. 4.9 ± 2.6, *p* = 0.18), number of previous non-implanted embryos (mean ± SD, 6.4 ± 3.6 vs. 7.5 ± 4.2, *p* = 0.06), and infertility etiology (11% vs. 10.2% with both female and male infertility, *p* = 0.9; 24.5% vs. 20.3% with male infertility, *p* = 0.6; 45.2% vs. 44.1% with female infertility, *p* = 0.9; 19.4% vs. 25.4% with idiopathic infertility, *p* = 0.35) were comparable in the cET and control groups (Table 1). The proportion of patients who benefited from oocyte/embryo donation also was similar between groups (21.3% in the cET and 13.6% in the control group, *p* = 0.25). Moreover, the proportion of patients with delayed receptivity window (i.e., after LH + 6/LH + 7 or Pg + 5/Pg + 6) was similar between groups (81.9% vs. 86.6% in the cET and control group, *p* = 0.64). Specifically, among the 46 patients in a natural cycle of the cET group, endometrium was receptive at LH + 8 in 48%, at LH + 6/LH + 7 in 22%, and at LH + 9 in 30% (Fig. 4). Among the 73 patients on HRT who underwent cET, endometrium was receptive at Pg + 7 (29%) and Pg + 8 (54%), followed by Pg + 5/Pg + 6 (10%) and Pg + 9 (7%). Among the 38 patients on HRT with GnRHa who underwent cET, endometrium was receptive at Pg + 8 in 63%, at Pg + 5/Pg + 6 in 13%, at Pg + 7 in 13%, and at Pg + 9 in 11% (Fig. 4).

**Table 1:** Patients’ characteristics

	cET	Controls	*p* value
Number of patients	157	60	
Age (years)	37.2 ± 4.3	36.7 ± 4.5	ns
Number of previous failed attempts (FET, FTET)	4.4 ± 1.9	4.9 ± 2.6	ns
Number of previous non-implanted embryos (FET, FTET)	6.4 ± 3.6	7.5 ± 4.2	ns
Infertility etiology (% of patients):			
Both (female and male)	11	10.2	ns
Female	45.2	44.1	ns
Male	24.4	20.3	ns
Idiopathic	19.4	25.4	ns
Endometrial thickness (mm)	8.9 ± 1.6	8.6 ± 1.2	ns
Number of transferred embryos (per cycle)	1.4 ± 0.5	1.6 ± 0.7	0.04
Cleavage stage embryos transferred (% of patients)	50.6	59.5	ns
Blastocysts transferred (% of patients)	49.4	40.5	ns

Data are expressed as the mean ± SD

*cET* customized embryo transfer, *FET* fresh embryo transfer, *FTET* frozen-thawed embryo transfer, *ns* non-significant

The implantation rate per cycle (22.7% vs. 7.2%, *p* = 0.0001) was significantly higher in the cET than in the control group (Table 2). In the cET group, a total of 277 embryos were transferred (63 gestational sacs/277 transferred embryos), resulting in 50 live births. In the control group, 125 embryos were transferred (9 gestational sacs/125 transferred embryos), resulting in 5 live births (*p* = 0.0002) (Table 2). Similarly, the positive β-hCG and clinical pregnancy rates per patient were higher in the cET than in the control group (44.6% vs. 16.7%, *p* = 0.0001; and 38.8% vs. 15%, *p* = 0.0006). The live birth rate per patient was 31.8% in the cET and 8.3% in the control group (*p* = 0.0002), despite the fact that the mean (± SD) number of transferred embryos per cycle was smaller in the cET than in the control group (1.4 ± 0.5 vs. 1.6 ± 0.7, *p* = 0.04) (Table 1). At the time of data collection, seven pregnancies in the cET group (4.5%) were still ongoing. Therefore, the live birth rate was underestimated for the cET group. After the first attempt, the implantation and live birth rates after cET were 24.8% (56 gestational sacs/226 embryos transferred) and 31.2%, respectively. The pregnancy outcomes per cycle according to the stage of the transferred embryo (cleavage stage embryos vs. blastocysts) are provided in Supplementary Table S1. Whatever the embryo stage, the pregnancy rate per cycle was significantly higher in the cET compared with the control group.

**Table 2:** Pregnancy outcome in patients with RIF after cET according to the Win-Test results and after the classical procedure (controls)

	cET	Controls	*p* value
Pregnancy outcome/patient			
Number of patients	157	60	
Pregnancy rate (β-hCG+) (%)	70 (44.6)	10 (16.7)	0.0001
Clinical pregnancy rate (%)	61 (38.8)	9 (15)	0.0006
Ongoing pregnancy rate (%)	57 (36.3)	5 (8.3)	0.00002
Live birth rate (%)	50 (31.8)	5 (8.3)	0.0002
Pregnancy outcome/cycle			
Number of cycles	195	79	
Pregnancy rate (β-hCG+) (%)	75 (38.5)	10 (12.7)	0.00002
Clinical pregnancy rate (%)	61 (31.3)	9 (11.4)	0.0004
Ongoing pregnancy rate (%)	57 (29.2)	5 (6.3)	0.00001
Live birth rate (%)	50 (25.6)	5 (6.3)	0.0002
Implantation rate (%)	63/277 (22.7)	9/125 (7.2)	0.0001

Data are expressed as the mean ± SD. The number of patients and cycles are indicated, and the percentage of patients or cycles is between brackets

*cET* customized embryo transfer

## Discussion

The Win-Test allowed identification of the endometrial receptivity window within the implantation window. Whatever the cycle type (natural, HRT, HRT with GnRHa), the receptivity window duration was longer than 24 h and shorter than 48 h in 78.5% of patients. Duration was longer than 48 h in 12% and shorter than 24 h in 9.5% of patients. This can explain why in a sub-group of patients (12%) more than two endometrial biopsies were necessary to identify the receptivity window. No more than two endometrial biopsies were performed during the same cycle, mainly at 48 h of interval, because local injury caused by consecutive biopsies can affect the endometrial transcriptomic profile [22]. Nevertheless, none of genes that are affected by endometrial biopsy-induced local injury is included in the Win-Test gene panel. Previous studies suggested that the implantation window may last 48 h [23], 2 to 4 days [24], 4 days [25], or 3 to 6 days [26]. Here, we showed that the receptivity window lasts about 2 days in most patients (~ 78%). This information is crucial and must be taken into account for cET of cryopreserved embryos. Our data from patients with three or more endometrial biopsies during at least two identical cycles suggest that the acquisition of the endometrial receptivity phenotype is a progressive process during both HRT and natural cycles. This notion is reinforced by the fact that the decidualization, essential for the acquisition of the endometrial receptivity phenotype, is also a progressive process that starts during the postovulatory phase (early-secretory phase) and culminates during the mid-secretory phase [14]. Conversely, the implantation window closes very rapidly, within 24 h after the occurrence of the receptivity window. In addition, we found that the receptive status timing is specific to each patient. According to the usual strategy for frozen-thawed blastocyst transfer during natural cycles, endometrium receptivity is considered to occur at LH + 6/LH + 7 [27–29]. Here, we showed that only 30% of the 46 patients with RIF who were screened with the Win-Test at these specific times had a receptive endometrium. In the 57 patients in a natural cycle in whom endometrium receptivity could be identified, the majority had a receptive endometrium at LH + 8 (42%) and LH + 9 (33.5%), and only 24.5% at LH + 6/LH + 7. During HRT cycles, blastocysts are usually transferred at Pg + 5/Pg + 6 [28–33]. At this time, only 25% of patients with RIF were identified as receptive, whereas 31% were receptive at Pg + 7 and 46% at Pg + 8. The others were receptive at Pg + 5/Pg + 6 (14%) and at Pg + 9 (9%).

These findings indicate that in most of our patients with RIF, the receptivity window was delayed by 1 to 2 days in natural cycles, and between 1 and 3 days during HRT, with or without GnRHa, reinforcing the hypothesis that the acquisition of the endometrial receptivity phenotype is slower in HRT than in natural cycles. This delay is consistent with previous reports suggesting that the endometrium of patients with RIF tends to be pre-receptive at Pg + 5 [34–36]. At Pg + 5/Pg + 6, the receptivity window was delayed in ~ 80% of our patients with RIF compared with 26% (*n* = 85 patients with RIF) and 47% (*n* = 62 patients with RIF) in the studies by Ruiz-Alonso et al. [34] and Tan et al. [36], respectively. In these two studies, endometrial receptivity was tested at Pg + 5 using a different assay, and RIF was defined in a different manner in the study by Tan et al. [36]. Conversely and differently from other reports [34], we never observed early endometrial receptivity occurrence during HRT and natural cycles. Our results are in accordance with the study by Bassil et al. [37] showing that only 35% of 41 patients with 0–2 previous failed embryo transfers were receptive at Pg + 5 (HRT cycles). These findings strongly suggest that the delay observed in patients in HRT cycles is a specific feature of HRT protocols and not of patients with RIF, contrary to what was suggested by other authors [38, 39]. Moreover, we previously reported differences in gene expression profile during the implantation window between HRT and natural cycles [40]. Indeed, in most patients, a minimum time of progesterone treatment seems to be necessary for endometrium maturation and for receptivity acquisition. Indeed, whatever the hormonal treatment, endometrium was receptive after 7 or 8 days of progesterone administration in patients with RIF. These results are consistent with the study by Prapas et al. [25] showing a significant higher pregnancy rates when day 2 embryos (4–6 cells) were transferred at day 4 or 5 after initiation of progesterone compared with day 2 or 3. In addition, as reported in the present study, endometrium receptivity occurrence time and duration are patient dependent, reinforcing the notion that the identification of the optimal timing for embryo transfer for each patient is essential to optimize ART effectiveness and to achieve a successful pregnancy. Indeed, endometrial receptivity timing varies among patients with similar characteristics (age, infertility etiology) and the same substitutive treatment (similar dose of estrogen and progesterone). Furthermore, some patients in HRT cycles who were evaluated with the Win-Test at more than 1 year of interval showed a similar endometrial profile, suggesting low intra-patient variability (data not shown).

The clinical relevance of the Win-Test was evaluated by performing cET according to the Win-Test results (*n* = 157 patients with RIF) or classical embryo transfer (*n* = 60 patients with RIF). The two groups were comparable in age, number of previous failed attempts, number of previous non-implanted embryos, and endometrial thickness during the periovulatory period. The fertility outcomes for patients who underwent conventional embryo transfer were consistent with previously published data showing lower pregnancy and implantation rates in women with RIF than without RIF [39]. Comparison of the two groups highlighted significantly higher pregnancy, clinical pregnancy, and live birth rates per patient in the cET than in the control group. Whatever the stage of the transferred embryos (cleavage stage embryos vs. blastocysts), the Win-Test strategy improved pregnancy outcomes despite the greater mean number of transferred embryos per cycle in the control group. These findings are very encouraging, and the Win-Test interest is currently investigated in a large cohort of patients without RIF.

This was the first prospective study reporting the Win-Test clinical effectiveness as a diagnostic tool to identify the receptivity window and to perform, in a subsequent identical cycle, cET according to the Win-Test results to improve the embryo-endometrial synchronization. The other strengths of the present study are the larger cohort of patients with RIF compared with previous studies [34, 37, 38, 41, 42], and the presence of a control arm to compare pregnancy outcomes among RIF patients who did and did not undergo cET after the Win-Test. The heterogeneity of protocols (natural and HRT ± GnRHa cycles) used for endometrial preparation is a limitation. However, the protocol remained the same for each patient during the assessment with the Win-Test and embryo transfer.

## Conclusion

By determining the specific cycle day within the implantation window where endometrium is receptive (i.e., receptivity window), the Win-Test showed that both the occurrence time and duration of the receptivity window are patient dependent during natural and HRT cycles. Moreover, the Win-Test highlighted that implantation failure could be partly due to inadequate timing of embryo transfer, resulting in embryo-endometrial desynchronization. Consequently, cET after the Win-Test to assess the endometrial receptivity status improves implantation, pregnancy, and live birth rates in patients with RIF.

## Electronic supplementary material

Supplementary Table S1 Pregnancy outcome per cycle in patients with RIF after (cET) or not (Controls) customized embryo transfer according to the Win-Test results in function of the stage of the transferred embryos: (a) early embryos, and (b) blastocysts. cET, customized embryos transfer (TIF 19.3 mb)

Supplementary Fig. S1 ROC analysis for the prediction of pregnancy outcome (positive β-hCG) using the mean transcript levels of the Win-Test genes (PNG 4948 kb)

High Resolution Image (TIF 19.3 mb)

Supplementary Fig. S2 The implantation window: a gradual opening and quick closure. The endometrial receptivity status was assessed using the Win-Test at different time points during natural or HRT cycles, as indicated. The figure shows the endometrial receptivity status profile of four patients during HRT and four patients during natural cycles. NR, non-receptive; PR, partially receptive; R, receptive; LH, luteinizing hormone; Pg, progesterone (PNG 4948 kb)

High Resolution Image (TIF 19.3 mb)
