# Supplementary figures and images for: Customized Frozen Embryo Transfer after Identification of the Receptivity Window with a Transcriptomic Approach Improves the Implantation and Live Birth Rates in Patients with Repeated Implantation Failure
Source: Reprod Sci. 2020 Jul 28;28(1):69–78. doi: 10.1007/s43032-020-00252-0 (PMC7782404; doi:10.1007/s43032-020-00252-0)

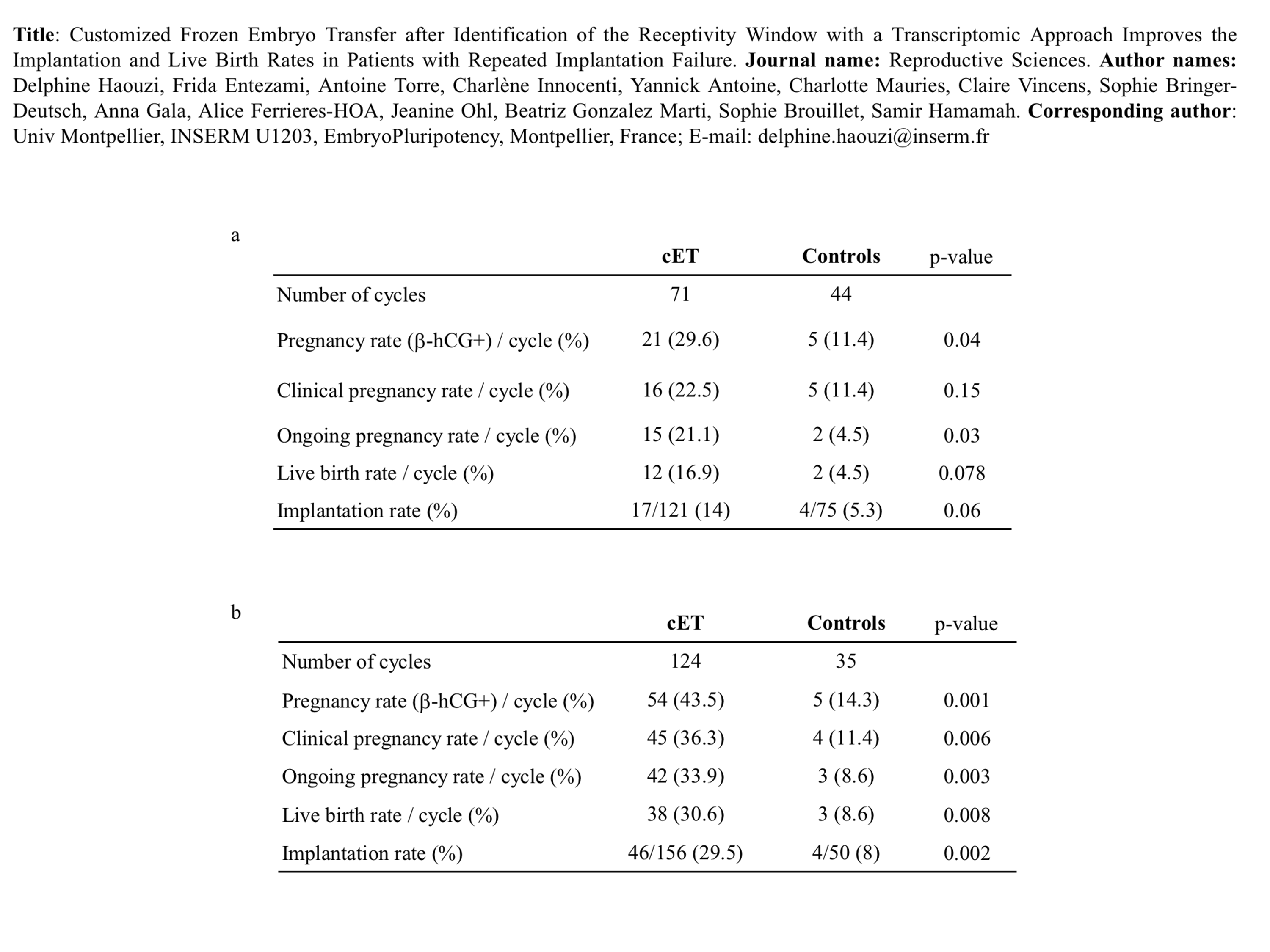

Supplement: Supplementary file 1 — Pregnancy outcome per cycle in patients with RIF after (cET) or not (Controls) customized embryo transfer according to the Win-Test results in function of the stage of the transferred embryos: (a) early embryos, and (b) blastocysts. cET, customized embryos transfer (TIF 19.3 mb) [file 43032_2020_252_MOESM1_ESM.tif]

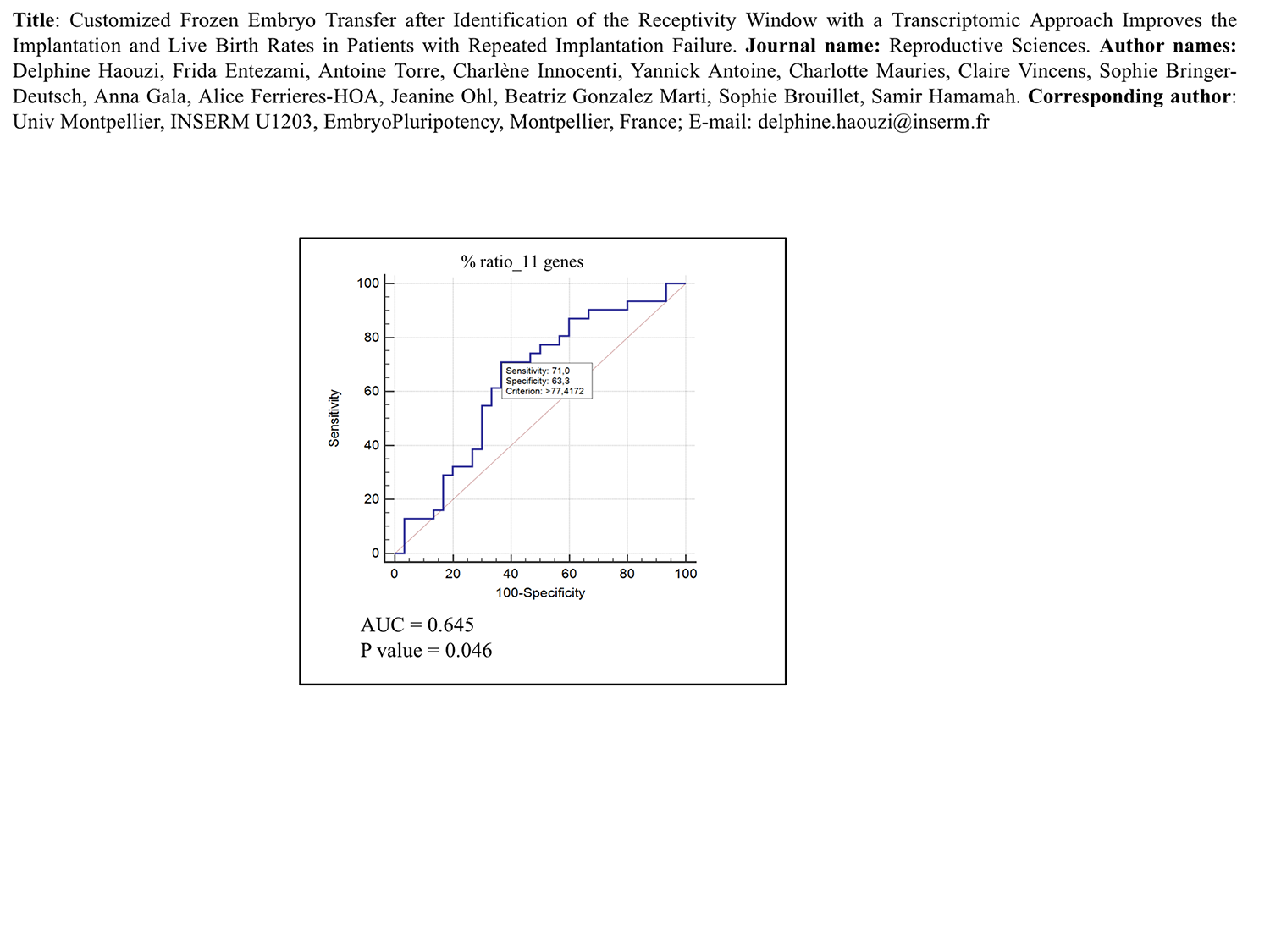

Supplement: Supplementary file 2 — ROC analysis for the prediction of pregnancy outcome (positive β-hCG) using the mean transcript levels of the Win-Test genes (PNG 4948 kb) [file 43032_2020_252_Fig5_ESM.png]

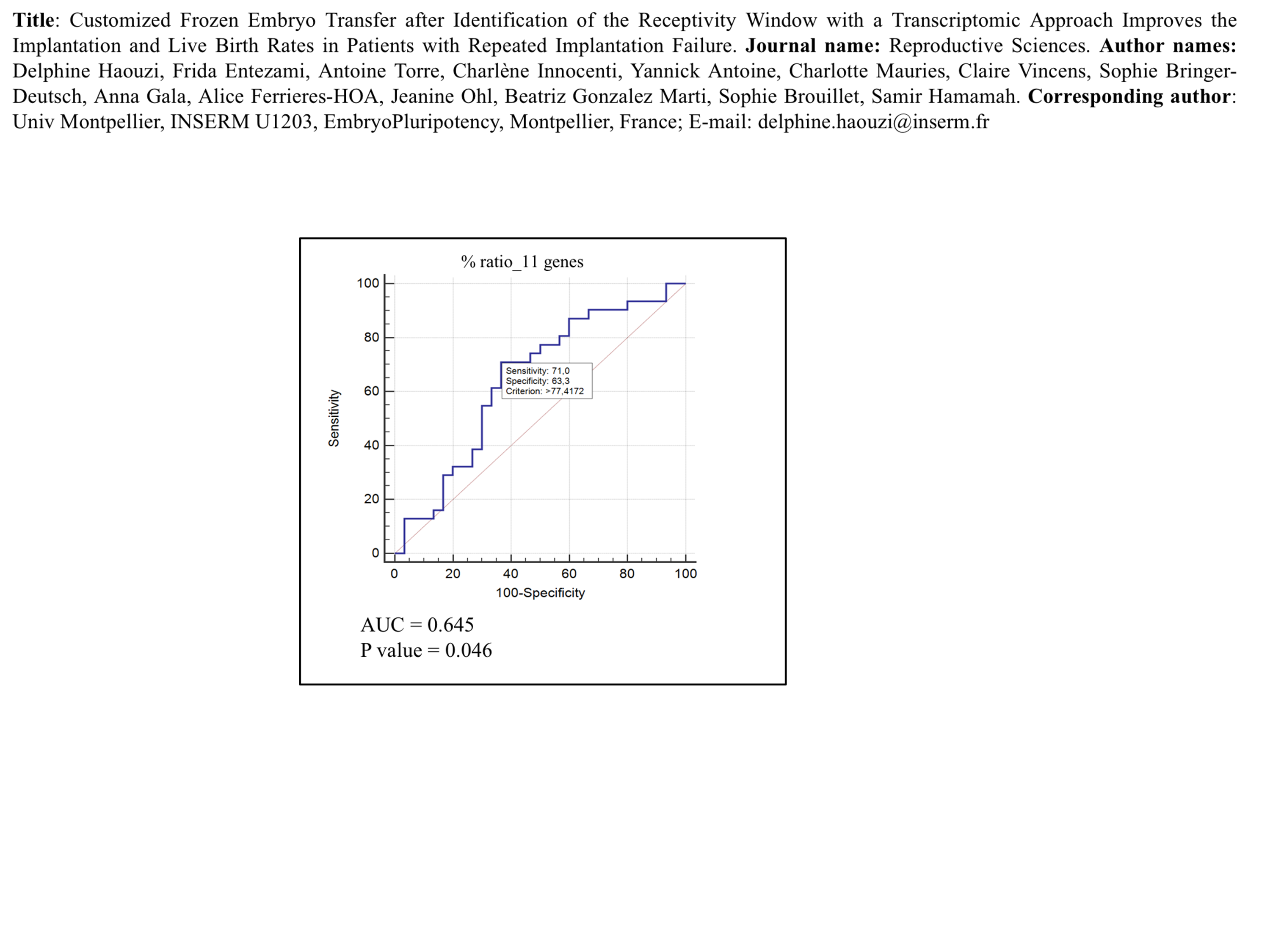

Supplement: Supplementary file 3 — High Resolution Image (TIF 19.3 mb) [file 43032_2020_252_MOESM2_ESM.tif]

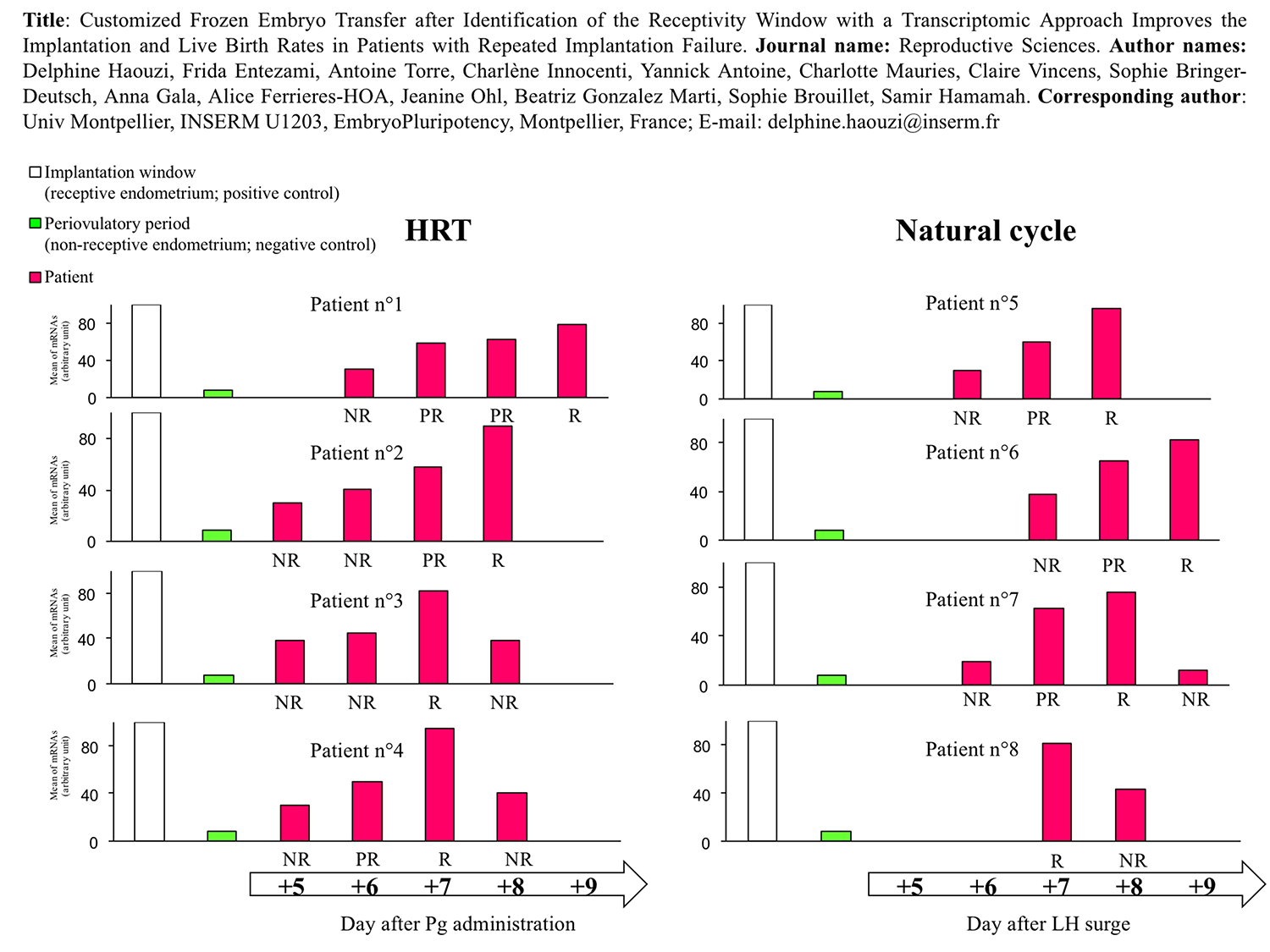

Supplement: Supplementary file 4 — The implantation window: a gradual opening and quick closure. The endometrial receptivity status was assessed using the Win-Test at different time points during natural or HRT cycles, as indicated. The figure shows the endometrial receptivity status profile of four patients during HRT and four patients during natural cycles. NR, non-receptive; PR, partially receptive; R, receptive; LH, luteinizing hormone; Pg, progesterone (PNG 4948 kb) [file 43032_2020_252_Fig6_ESM.png]

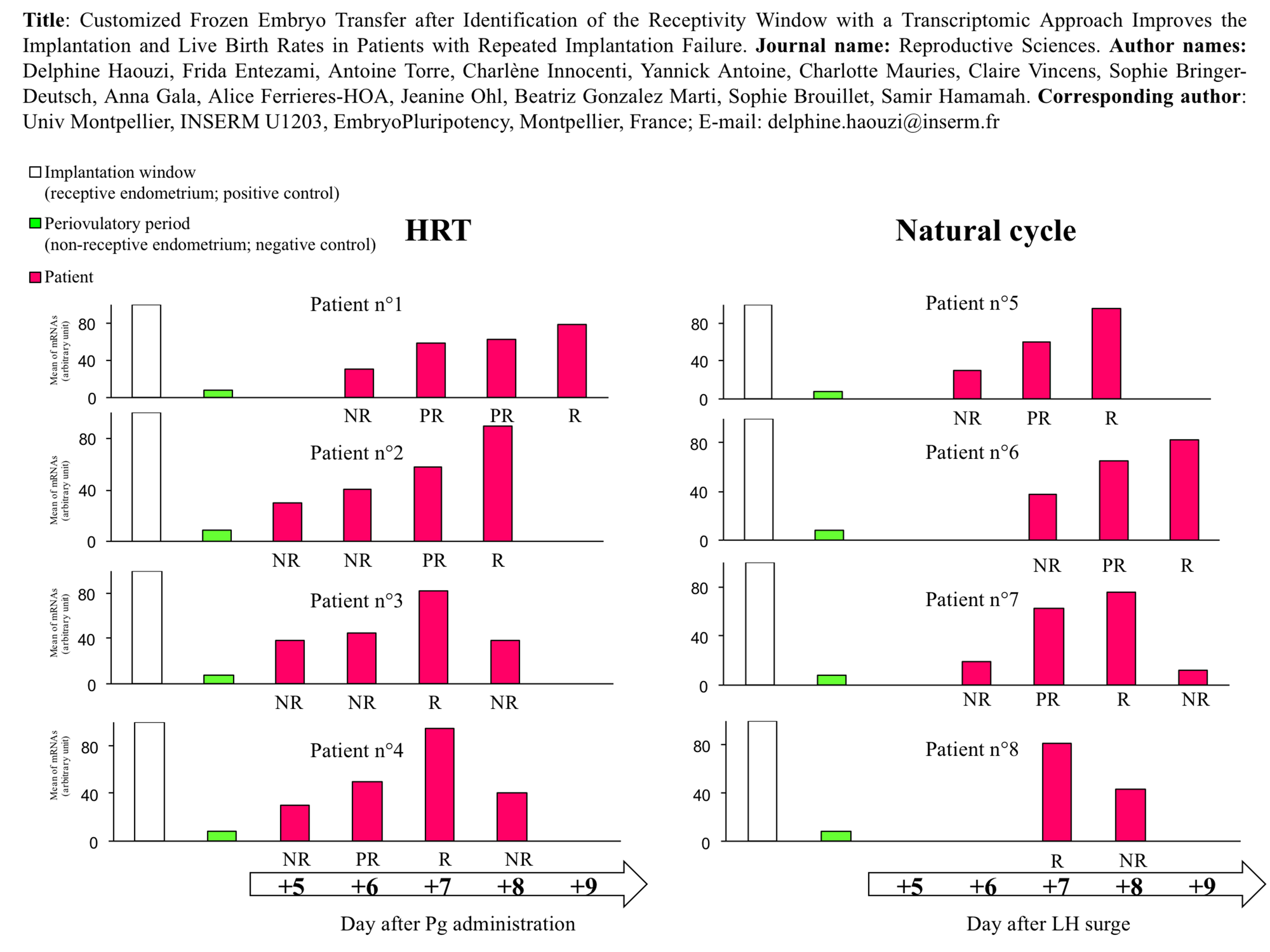

Supplement: Supplementary file 5 — High Resolution Image (TIF 19.3 mb) [file 43032_2020_252_MOESM3_ESM.tif]
